# A Species-Specific Strategy for the Identification of Hemocoagulase *Agkistrodon halys pallas* Based on LC-MS/MS-MRM

**DOI:** 10.3389/fmolb.2022.831293

**Published:** 2022-05-30

**Authors:** Ruiqing Xian, Congcong Wang, Liping Gong, Baojian Hang, Weijian Wang, Xunjie Zhang, Hongmin Du, Fengshan Wang, Feng Shi

**Affiliations:** ^1^ Biological Products Inspection Division, Shandong Institute for Food and Drug Control, Jinan, China; ^2^ National Medical Product Administration (NMPA) Key Laboratory for Research & Evaluation of Genetic Drugs, Jinan, China; ^3^ Key Laboratory of Chemical Biology (Ministry of Education), Institute of Biochemical and Biotechnological Drugs, School of Pharmaceutical Sciences, Cheeloo College of Medicine, Shandong University, Jinan, China; ^4^ R&D Department, Avanc Pharmaceutical Co., Ltd., Jizhou, China

**Keywords:** hemocoagulase, snake venom thrombin-like enzymes, marker peptide, mass spectrometry, proteomics, MRM

## Abstract

Hemocoagulase *Agkistrodon halys pallas* is a complex mixture composed of snake venom thrombin-like enzymes (svTLEs) and small amounts of thrombokinase-like enzymes. It has been widely used as a hemostatic with rapidly growing marketing due to its advantage of localized clotting fibrinogen other than systemic coagulation. However, svTLEs from different species have various structures, functions, and hemostatic mechanisms. To ensure the efficacy and safety of Hemocoagulase *Agkistrodon halys pallas*, an exclusive and sensitive method has been developed to identify specific marker peptides based on liquid chromatography-tandem mass spectrometry with multiple reaction monitoring (LC-MS/MS-MRM) mode. By combining transcriptomics and proteomics, a series of species-specific peptides of *Agkistrodon halys pallas* were predicted and examined by LC-MS/MS. After reduction, alkylation, and tryptic digestion were performed on Hemocoagulase *Agkistrodon halys pallas*, a target peptide TLCAGVMEGGIDTCNR was analyzed by LC-MS/MS-MRM. It offers a new and effective approach for the quality control of Hemocoagulase *Agkistrodon halys pallas* products. This method is superior to the current assays in terms of sensitivity, specificity, precision, accuracy, and throughput. The strategy can also be applied in studying other important protein-based medicines.

## Introduction

Snake venom thrombin-like enzymes (svTLEs) are members of snake venom serine proteinases (SVSPs) which have the activity of arginine esterase and acylamidase (Ullah et al., 2018). svTLEs are used as hemostatic drugs, defibrinogenating agents, and antithrombotic and diagnostic agents in clinics because of its ability of clotting fibrinogen (Waheed et al., 2017). In addition, some svTLEs can also activate plasminogen and induce platelet aggregation (Castro et al., 2004; Sajevic et al., 2011). Hemocoagulase atrox for Injection (Reptilase^®^ and Baquting^®^) isolated from the venom of *Bothrops atrox* is the earliest and most widely used hemostatic in clinical settings (Funk et al., 1971; Li et al., 2019). Hemocoagulase Agkistrodon for Injection (Suling^®^) is isolated from the venom of *Deinagkistrodon acutus* and contains a single thrombin-like component (Li et al., 2018). Hemocoagulase for Injection (Sulejuan^®^) is isolated from the venom of *Vipera russelli siamensis* which is composed of batroxobin and active factor X (FXa). The active ingredients of Hemocoagulase *Agkistrodon halys pallas* for Injection (Bangting^®^) are thrombin-like enzymes and thrombokinase-like enzymes. The amino acid sequences, glycosylation sites, structures, and hemostatic mechanisms of svTLEs from different species are a little different. Hemocoagulase *Agkistrodon halys pallas* acts on fibrinogen, which first releases fibrinopeptide B and then releases fibrinopeptide A after a period. The rest act only on the fibrinogen Aα-chain (FPA) (Guan et al., 1984; Zeng et al., 2013). The present qualitative assays of hemocoagulase (including biochemical reaction, chemical chromogenic reaction, liquid chromatography-based peptide mapping, electrophoresis method, and activity measurements) are not accurate enough and the species cannot be identified. Establishing a new strategy to identify specific species is necessary to strengthen quality control and promote the safety of drug use in clinics.

Mass spectrometry plays a key role in the structural characterization of glycans, proteins, glycan–protein interactions, and protein–protein interactions (Britt et al., 2022; Grabarics et al., 2022; Sheng et al., 2021; Zhang and Chi, 2021). Shotgun proteomics based on liquid chromatography−tandem mass spectrometry (LC−MS/MS) and database search are widely used for the qualitative and quantitative analysis of proteins in complex biological samples (Huang and Wang, 2021; Marques et al., 2021). Database search is based on a database of known protein sequences in genomics. Proteogenomics, a combination of proteomics and genomics, is an efficient strategy to detect new proteins and peptides (Lu et al., 2019; Yang et al., 2021). The database derived from transcriptome sequencing was used for the matching of MS/MS data and the proteomic assays were used for the verification of new protein sequences. The development of proteomics and genomics plays a key role in clinical diagnosis and treatment, identifying potential biomarkers, discovery of drug targets, and mechanisms of disease development (Bai et al., 2021; Wang et al., 2021; Yang et al., 2021). In the global healthcare emergency caused by SARS-cov-2 since 2020, genomics, proteomics, and glycoproteomics have played a key role in the development of nucleic acid test kits, variant identification, vaccine development research, developing treatment options, studying infection mechanisms, and biomarkers’ investigation (Alaiya et al., 2021; Wang L. et al., 2021).

Multiple-reaction monitoring (MRM), involving the detection of targeted ion pairs, is a sensitivity and high specificity MS technique and has been extensively used in small molecules and targeted proteomic assays (Shi et al., 2014; Li et al., 2017; Zhang et al., 2020). Identification and validation of unique prototypic peptides by omics technologies and then quantification by liquid chromatography-tandem mass spectrometry-multiple reaction monitoring (LC-MS/MS-MRM) have been widely reported (Zhang et al., 2019; Dong et al., 2021; Suvarna et al., 2021). In an early study, two LC-MS/MS-MRM methods have been built to analyze traditional Chinese medicines, Colla corii asini (CCA) and Compound Xiling Jiedu Capsules (Li et al., 2017; Wang Y. T. et al., 2021). Based on the species-specific peptides, different species of collagen can be identified such as those of a donkey, horse, cattle, and pig. For Compound Xiling Jiedu Capsules, the antelope horn and buffalo horn can also be identified by the LC-MS/MS-MRM method. In this study, a series of specific peptides in Hemocoagulase *Agkistrodon halys pallas* has also been identified by proteomics. Based on the species-specific peptide, a method for the assay of Hemocoagulase *Agkistrodon halys pallas* was established using LC-MS/MS-MRM. Compared with previous methods, the new method has several advantages including faster analysis and better specificity, accuracy, and efficiency.

## Materials and Methods

### Chemicals and Reagents

Trypsin (sequencing grade) was purchased from Sigma (United States). Ultrafiltration units with a 3 kDa molecular weight cut-off (MWCO) were purchased from Sartorius (Germany). Guanidine hydrochloride, DL-dithiothreitol (DTT), and iodoacetamide (IA) were purchased from VETEC (United States). Formic acid, acetonitrile [high-performance liquid chromatography (HPLC)-grade] and water (HPLC-grade) were purchased from Thermo Fisher Scientific (United States). All the other chemicals and reagents were of analytical reagent grade or better in the commercial market. Marker peptide (TLCAGVMEGGIDTCNR) standard was synthesized by ChinaPeptides (China). Venom gland and Hemocoagulase *Agkistrodon halys pallas* stocks were purchased from Avanc Pharma (China). The lyophilized powders of the snake venom of *Vipera russelli siamensis*, *Bothrops atrox*, and *Deinagkistrodon acutus* were purchased from Avanc Pharma (China).

### Construction of the Transcriptome Protein Database

The venom of the snake was drained, then the venom glands of the snake were carefully separated, and the attached connective tissue was carefully dissected. The gland was cut into small pieces and rinsed with precooled PBS solution quickly. After quick-freezing in liquid nitrogen for 1–3 h, the sample was transferred to −80°C dry ice for storage and transportation. The extraction of total RNA was performed by the TRIZOL method according to the manufacturer’s protocol. Second and third-generation sequencing was performed using Illumina NovaSeq6000 and PacBio Sequel II, respectively. The clean data were obtained after filtering and other steps of raw data. Then, the open reading frame (ORF) was analyzed for gene analysis. After the determined transcript, the ORF sequence was translated into the corresponding protein sequence, and used as a transcriptome database for further mass-spectrometry database search.

### LC-MS/MS Shotgun Analysis

Hemocoagulase *Agkistrodon halys pallas* was resolved in denaturing buffer (0.5 M Tris-HCl, 2.75 mM EDTA, 6 M guanidine hydrochloride, pH 8.1). Then the samples were reduced and alkylated. The reaction reagents were removed by ultrafiltration (MWCO 3 kDa). The samples were digested with trypsin at an enzyme-to-protein ratio of 1/50 (w/w) at 37°C overnight, and then the peptides were recovered by ultrafiltration (MWCO 3 kDa). Nano-LC-MS/MS analysis was performed by using a Thermo Easy-nLC and Orbitrap Fusion mass spectrometer equipped with a nano-spray ion source. The Acclaim PepMap^®^100 C18 trapping column (100 μm × 2 cm) and Acclaim PepMap^®^100 C18 analytical column (75 μm × 15 cm) were used to separate the peptides. Mobile phase A was 0.1% formic acid in 2% acetonitrile and mobile phase B was 0.1% formic acid in 98% acetonitrile. A step gradient of B 0–7% for 20 min, 7%–22% for 60 min, 22%–35% for 20 min, and 35%–90% for 10 min was used. The flow rate was set at 0.3 μl/min.

The database search was performed by the Proteome Discoverer 2.1 (Thermo Fisher Scientific). The general settings are as follows: the mass analyzer was orbitrap, the activation type was high energy collision dissociation (HCD), the polarity mode was positive, and the databases were the UniProt thrombin-like enzyme (TLE) database and transcriptome protein database. The enzyme was trypsin, precursor mass tolerance was 10 ppm, fragment mass tolerance was 0.02 Da, dynamic modifications (peptide terminus) were oxidation/+15.995 Da (M), dynamic modifications (protein terminus) were acetyl/+42.011 Da (N-terminus) and carbamido methyl/+57.021 Da (C), and the peptide confidence at least was settled as high. High confidence identification of proteins was ensured by setting the target FDR (strict) at 0.01. After the database search, the TLE sequence of *Agkistrodon halys palla*s was identified from the transcriptome protein database. The TLEs of *Deinagkistrodon acutus* and *Bothrops atrox* were downloaded from the UniProt (https://www.uniprot.org/, Q9I8X2 and P04971). The unique amino acid residues of *Agkistrodon halys pallas* were spotted using the software BioEdit Sequence Alignment Editor. Combined with database searching results, the suitable marker peptide was screened.

### LC-MS/MS MRM Analysis

The LC-MS/MS MRM analysis was performed using the AB SCIEX QTRAP^®^ 6500 LC-MS/MS System equipped with an electrospray ionization (ESI) source (AB Sciex, United States). An ACQUITY UPLC BEH C18 (1.7 μm, 2.1 mm × 50 mm) column was used with the mobile phase A of 0.1% formic acid in water and mobile phase B of 0.1% formic acid in acetonitrile. The gradient was mobile phase B 20% for 1 min, 20%–90% for 4 min, 90%–90% for 2 min, and 20% for 1 min. The flow rate was set at 0.2 ml/min. The auto-sampler and the column temperature were maintained at 8°C and 40°C, respectively. The mass spectrometer was in the positive MRM mode and the temperature of the ion source was set at 500°C. The nebulizing gas and drying gas were set at 50 and 60 psi, respectively. The collision energy and declustering potential for the marker peptide were optimized using the peptides synthesized correspondently.

### Optimization of Digestion Parameters

To maximize the market peptide released from the sample, the final concentration of the DTT, enzyme-protein ratio, and enzyme digestion time were optimized. The standard protocol was as follows: the 600 μl Hemocoagulase *Agkistrodon halys pallas* stock (0.3 μg/μl) was diluted to 10 ml by 25 mmol/L ammonium bicarbonate solution. The 200-μl diluent was reduced by 10 μl 0.2 mol/L DTT and incubated for 1 h at 60°C. After that, 20 μl of 0.1 mol/L IA was added and incubated for 30 min at room temperature in the dark. Then, 25 mmol/L ammonium bicarbonate was added to dilute the sample by ten times, and 4 μg trypsin was added and incubated for 30 min at 37°C.

### Validation of the HPLC-MRM-MS Method

The method for the quantitative analysis of the proposed hemocoagulase was validated in specificity, linearity, limits of detection (LOD), limits of quantification (LOQ), precision, repeatability, recovery, and stability. The sample solution (SS) of Hemocoagulase *Agkistrodon halys pallas* was prepared by the optimized pretreatment methods. The blank sample (BS) was 25 mmol/L ammonium bicarbonate solution and the control sample (CS) was a synthetic thrombin-like enzymes peptide (STLEP). The other snake venom samples (OSVS) were dissolved to 1 mg/ml using 25 mmol/L ammonium bicarbonate solution. The aforementioned solutions were treated in the same way as described in the Optimization of digestion parameters section. The specificity of the method was evaluated by detecting no peaks at the same retention time in the BS compared with the SS, CS, and OSVS. The CS containing the reference standards of STLEP was prepared and diluted to a series of appropriate concentrations for the LC-MS/MS-MRM analysis. The calibration curves were constructed by plotting the peak area (Y-axis) against the final concentrations of STLEP (X-axis). The LOD and LOQ were determined at a signal-to-noise ratio (S/N) of at least 3 and 10 under the same chromatographic conditions. The solutions of the standards were analyzed for six replicates for the precision test. For the repeatability test, six sample solutions were prepared in parallel for analysis. Stability was assessed by repeatedly determining the SS which was kept in the auto-sampler (8°C) for 0, 2, 4, 8, 10, and 24 h. The accuracy of the method was evaluated by the recovery test. The fixed STLEP content added in the SS was detected and compared with the known content. The spike recovery test was performed using three concentration levels and each set of addition was repeated three times. The mean recovery percentage was calculated by the formula:

Recovery (%)=(observed amounts−οriginal amounts)/spiked amounts × 100%.

## Results

### Study Design

**FIGURE 1:**
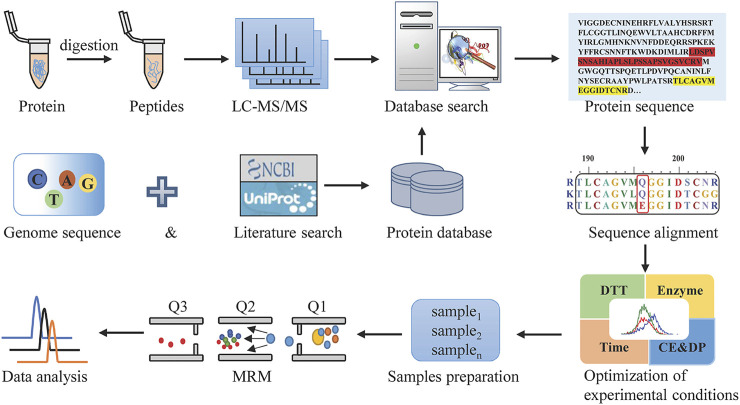
Workflow to discover the marker peptides for analyzing Hemocoagulase *Agkistrodon halys pallas*.

MRM, as the most commonly used targeted proteomics assays, is a sensitive and exclusive method to conduct the qualitative and quantitative analysis of known components in a sample with a complex matrix. For Hemocoagulase *Agkistrodon halys pallas*, the sequence of this thrombin-like enzyme is unattainable. In this study, a combination of transcriptomics and shotgun proteomics assays was built, with the overall workflow shown in Figure 1. The transcriptomics database by transcriptome sequencing and the UniProt database which contained snake thrombin-like enzyme sequences were used as the comprehensive protein database. Then after reduction, alkylation, and digestion of the sample, raw data were obtained by LC-MS/MS analysis. After the database search, the thrombin-like enzyme of *Agkistrodon halys pallas* was identified by high-quality interpretation of MS/MS data. By sequence alignment of theoretical sequences, the unique amino acid residues of *Agkistrodon halys pallas* were spotted and a suitable species-specific peptide was screened. Finally, after method development and validation, a highly specific, accurate, and efficient LC-MS/MS-MRM was established.

### Shotgun Proteomics Assays

**FIGURE 2:**
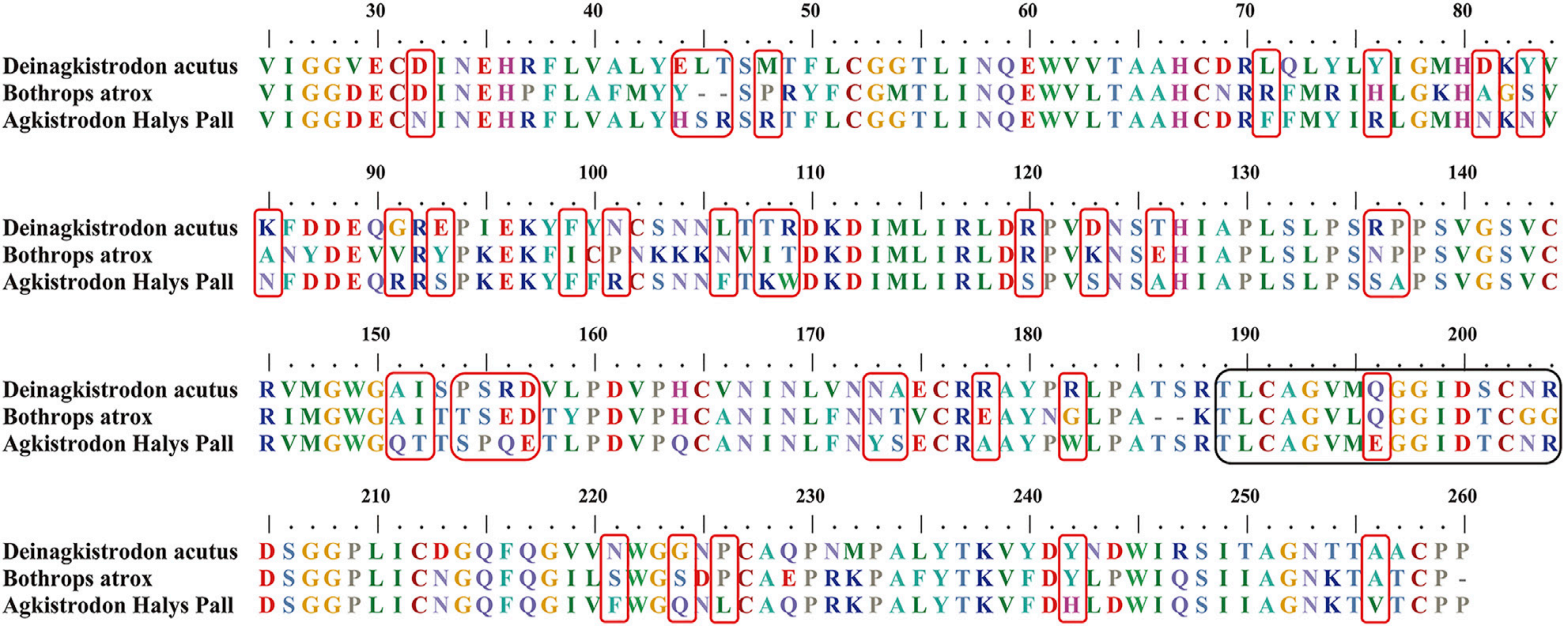
Partial sequence alignments of snake venom thrombin-like enzymes in *Deinagkistrodon acutus*, *Bothrops atrox*, and *Agkistrodon halys pallas*. The unique amino acid residues for *Agkistrodon halys pallas* are indicated in red boxes. The theoretical tryptic marker peptide which contains the unique amino acid residues studied in this work is in black boxes.

**FIGURE 3:**
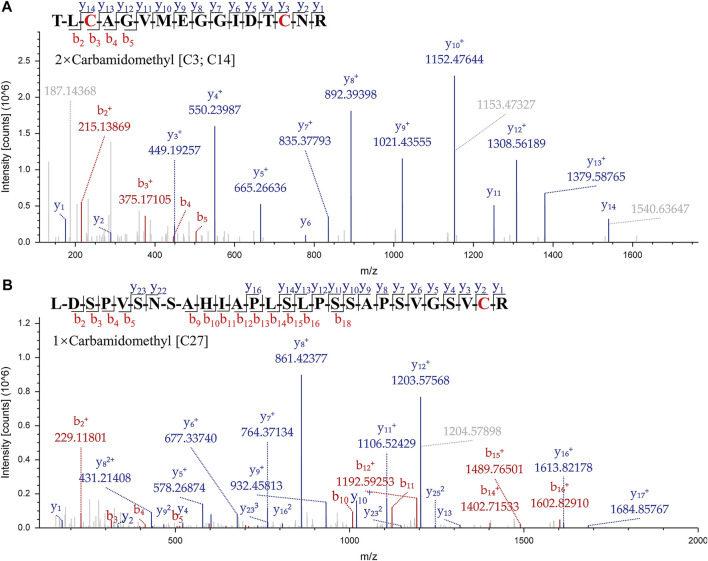
MS/MS spectrums of the marker peptides for *Agkistrodon halys pallas*. **(A)** Peptide TLCAGVMEGGIDTCNR, *m/z* = 877.39, double charged. **(B)** Peptide LDSPVSNSAHIAPLSLPSSAPSVGSVCR, *m/z* = 935.81, triple charged.

A suitable marker peptide is critical in developing an LC-MS/MS method for species-specific identification and quantification. After pretreatment of the Hemocoagulase *Agkistrodon halys pallas*, a shotgun proteomics analysis by nano-LC-MS/MS was performed to find the MS evidence. When the UniProt thrombin-like enzyme database was used, the results of Proteome Discoverer 2.1 showed that the thrombin-like enzyme kangshuanmei (P85109) and snake venom serine protease salmobin (O73800) have high sequence coverage, which were 65% and 53%, respectively. In the absence of the thrombin-like enzyme of *Agkistrodon halys pallas* in the UniProt database, the transcriptomics database was obtained by mRNA sequencing and the full-length translated mRNA-seq of the venom gland. After raw data were analyzed by Proteome Discoverer 2.1, the TLE sequence of *Agkistrodon halys pallas* can be identified from the transcriptomics database, with a 99% sequence coverage. The transcript sequences and other available svTLE sequences from different species were aligned and compared using the BioEdit Sequence Alignment Editor software. The partial sequence alignments of svTLEs of *Agkistrodon halys pallas*, *Deinagkistrodon acutus*, and *Bothrops atrox* are shown in Figure 2 as an example. The identical amino acid residues among different species are represented in the same color, and discrepant amino acid residues are represented in different colors for easy identification. Thirty-eight amino acid residues located within this sequence were not identical compared with the other two species. The corresponding peptides containing these amino acid residues were analyzed to find the marker peptides. Four potential species-specific marker peptides of *Agkistrodon halys palla*s were found: ^94^LDSPVSNSAHIAPLSLPSSAPSVGSVCR^121^, ^122^VMGWGQTTSPQETLPDVPQCANINLFNYSECR^153^, ^165^TLCAGVMEGGIDTCNR^180^, and ^181^DSGGPLICNGQFQGIVFWGQNLCAQPR^207^. The blast search revealed that these four peptides were unique in the UniProt database. The presence of these four theoretical marker peptides in Hemocoagulase *Agkistrodon halys pallas* was also verified by LC-MS/MS as shown in Figure 3. The marker peptides for hemocoagulase were selected based on three criteria. First, the peptide can be detected consistently in both hemocoagulase substances and drug products. Second, the peptide should have a suitable length to make it feasible for the MRM method. Third, the signal intensity of the peptide should match the spectrum of the peptide. Considering the long peptides were difficult and costly to be synthesized, the peptide ^165^TLCAGVMEGGIDTCNR^180^ was selected as the marker peptide for quantification.

### Validation of the Marker Peptide and Optimization of LC-MS/MS-MRM

**FIGURE 4:**
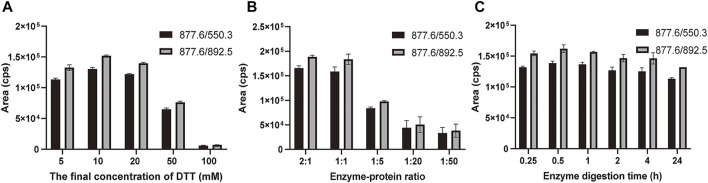
Optimization of digestion conditions. **(A)** Final concentration of DTT reduction (5, 10, 20, 50, and 100 mM) and alkylation with iodoacetamide (DTT-IA ration 1:2). **(B)** Enzyme-protein ratio (2:1, 1:1, 1:5, 1:20, and 1:50). **(C)** Enzyme digestion time (0.25, 0.5, 1, 2, 4, and 24 h) with trypsin at 37°C. Bars are mean ± SE (*n* = 3).

The marker peptides’ m/z was extracted in the raw data by Xcalibur software. The marker peptide (TLCAGVMEGGIDTCNR) standard was synthesized to optimize the method. To develop a rapid method to identify the marker peptide, an LC-MS/MS-MRM method was used to detect the parent and product ions. This method is rapid and high-sensitive, with better results for low-concentration components. From the MS/MS spectrum of TLCAGVMEGGIDTCNR, 877.6 (2+) > 550.3 was chosen as the qualitative ion pair, and 877.6 (2+) > 892.5 as the quantitative ion pair. The optimized gradient and voltage are shown in Table 1. To optimize the digestion conditions, the following parameters were optimized: the final concentration of DTT (5, 10, 20, 50, and 100 mM), enzyme-protein ratio (2:1, 1:1, 1:5, 1:20 and 1:50), and digestion time (0.25, 0.5, 1, 2, 4 and 24 h). As shown in Figure 4, 877.6 (2+) > 892.5 exhibits a higher mass response than 877.6 (2+) > 550.3. When the final concentration of DTT is more than 20 mM, the signal response is significantly decreased. Hence, 10 mM was the optimal final concentration of DTT. A high enzyme-protein ratio (2:1, 1:1) was better in improving the digestion efficiency. To reduce the interference of impurities, 1:1 was chosen as the optimal enzyme-protein ratio. When the enzyme digestion time was changed from 0.25 to 24 h, the signal response was not significantly changed. For complete digestion, 0.5 h is enough.

**TABLE 1:** MRM transitions for the marker peptide of *Agkistrodon halys pallas.*

Sequence	Parent ion (*m/z*)	Product ion (*m/z*)	Collision energy (CE)	Declustering potential (DP)
TLCAGVMEGGIDTCNR	877.6 (2+)	550.3	60	135
		892.5	45	135

### Method Validation

**FIGURE 5:**
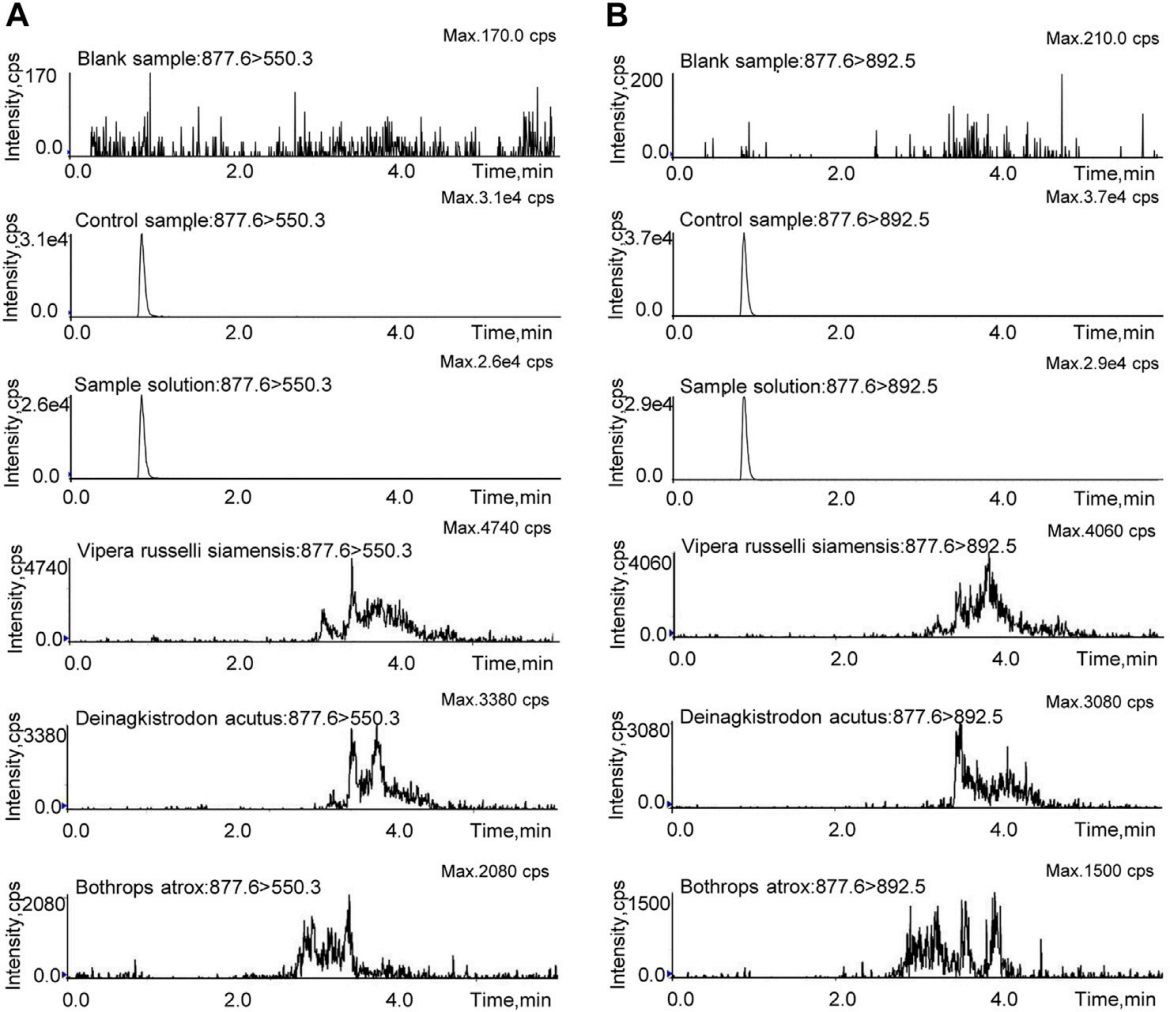
MRM investigation of TLCAGVMEGGIDTCNR in the blank sample, control sample, sample solution, and the other snake venom samples. **(A)** Qualitative ion pair (m/z = 877.6 > 550.3). **(B)** Quantitative ion pair (m/z = 877.6 > 892.5).

The LC-MS/MS-MRM method was validated in regard to specificity, linearity and sensitivity, accuracy and precision, recovery, stability, and repeatability. The blank sample, control sample, test sample solution, and the other snake venom samples were prepared with the same pretreatment method and then analyzed by LC-MS/MS-MRM. As shown in Figure 5, the marker peptide was detected in the test and control samples, but not in the blank sample. There were no interference peaks in the spectrum throughout the test. This method can distinguish the analyte from the matrix with good specificity. As for the snake venom of *Vipera russelli siamensis*, *Bothrops atrox*, and *Deinagkistrodon acutus*, in the spectrum, miscellaneous peaks appeared in other retention times with poor peak shape due to many interference factors in complex matrix samples. But there was no chromatographic peak at the same retention time. These results suggest that this method is highly species-specific and can identify *Agkistrodon halys pallas* from *Vipera russelli siamensis*, *Bothrops atrox*, and *Deinagkistrodon acutus.* The control solution was diluted into a series of appropriate concentrations and analyzed by the LC-MS/MS. The result shows the linear relationship between the peak area and the concentration of the marker peptide. The linear equations was y = 844208x -19,827. R^2^, with a coefficient of determination of 0.999. The LOD and LOQ were evaluated when the targeted peak intensity was at least 3 and 10 times higher than that in the matrix, which was 2 ng/ml and 6 ng/ml, respectively.

The precision of the method was evaluated by the relative standard deviation (RSD). The sample was injected into the HPLC-MS/MS six times continuously. The mean value was 14.53 μg/ml and the RSD was 2.1%, which shows the excellent precision of the method (Supplementary Table S1). The repeatability of the method was evaluated by preparing samples in sextuplicate. The RSD was 3.2% for the concentration level of 14.40 μg/ml (Supplementary Table S2). These results indicated a good reproducibility of pretreatment procedures. These precision and repeatability results were within the acceptable range, which means the method was precise and repeatable. Some samples may have a low signal or even no signal because of the matrix effect, or something in the matrix had a coincident peak with the targeted peptide, which would affect the accuracy of the result. Analyte recoveries can be used to assess the matrix effect. A certain amount of synthetic peptide was spiked into Hemocoagulase and then detected by LC-MS/MS-MRM. Recovery is defined as the ratio of the actual value of the synthetic peptide measured after the test to the theoretical amount. The quantitative ability of the method can be evaluated using the recovery and the RSD. As shown in Table 2, the recovery of three different concentrations of six replicates ranges from 86.0% to 100.7%. The mean recovery was 93.3% and the RSD was 6.3%, which was acceptable for peptide detection. Sample stability is critical for the long-term analysis of a large number of samples. The sample solution was kept at 8°C in the auto-sampler and injected for detection after 0h, 2h 4, 8, 10, and 24 h. The RSD of the peak area corresponding to the quantitative ion pair of the marker peptide was calculated and the result was <5%, indicating that the marker peptide in the solution was stable within 24 h at 8°C (Supplementary Table S3). The developed LC-MS/MS-MRM method was applied for the quantitative determination of three batches of Hemocoagulase *Agkistrodon halys pallas* (20190201, 20190202, and 20190203). The results showed that the contents of TLE were 15.33 μg/ml, 14.42 μg/ml, and 15.50 μg/ml, and there were no significant differences among the three batches (Supplementary Table S4).

**TABLE2:** Recovery test of the established LC-MS/MS-MRM method.

#	Original (ng)	Spiked (ng)	Found (ng)	Recovery (%)	RSD (%)
1	87	50	130	86.0%	6.3%
2			130	86.0%	
3			130	86.0%	
4		100	180	93.0%	
5			184	97.0%	
6			185	98.0%	
7		150	234	98.0%	
8			229	94.7%	
9			238	100.7%	

## Discussion

Hemocoagulase is one of the most important and indispensable drugs used in surgical operations. Hemocoagulase for Injection is a member of the biological product family, exhibiting a fine hemostasis and coagulation efficacy, which has been widely used as a hemostatic in China (Li et al., 2019). At present, the hemagglutinin drugs on the market in China are mainly isolated from the venom of *Bothrops atrox* (Baquting^®^), *Deinagkistrodon acutus* (Suling^®^), *Vipera russelli siamensis* (Sulejuan^®^), and *Agkistrodon halys pallas* (Bangting^®^), and their main active ingredients are svTLEs. However, svTLEs from different snake species vary in structures, hemostatic mechanisms, and pharmacological effects. Therefore, the accurate identification of snake venom species and the content of svTLEs is essential to ensure the quality of this product family. The existing quality control technology has been able to ensure the stability of the product quality, but the species cannot be identified. The identification and protein detection methods on snake venom species are mainly ELISA, liquid chromatography, and electrophoresis, which have disadvantages including complexity, low sensitivity, and poor specificity.

With the rapid development of biological mass spectrometry technology, mass spectrometry-based proteomics study is becoming increasingly mature. The proteomics technology based on peptide biomarkers provides a new idea for food and drug quality control (Huang and Wang, 2021; Marques et al., 2021). A marker peptide refers to an amino acid sequence which is unique for a protein and can be specifically distinguished from other proteins. Because of the differences in the DNA of different species, there should be differences in the amino acid sequences for proteins translated by DNA. Based on this principle, different peptides can be obtained after the hydrolysis of proteins by specific proteases, which can be used as characteristic markers for species identification or qualitative and quantitative analysis of target proteins.

The qualitative and quantitative analysis technology of proteins based on marker peptides has been successfully applied to the identification of meat products, the adulteration screening of traditional Chinese medicines, etc. However, there are few studies in the field of biochemical drugs, and there are no reports on the qualitative and quantitative study of snake venom blood coagulation based on marker peptides. In this study, the sequence of the pit viper thrombin-like enzyme was characterized by combining transcriptomics and proteomic approaches. By the means of bioinformatics, a series of potential marker peptides which can be distinguished from other snake species have been identified. After the method optimization, a rapid LC-MS/MS-MRM method for the qualitative and quantitative analysis of hemocoagulase was established. This study improved the quality standard of Hemocoagulase *Agkistrodon halys pallas*, which can ensure the safety and effectiveness of clinical medications. The study also provided a new strategy for the quality control of other snake venom products. However, as Hemocoagulase for injection contains a lot of gelatin and other ingredients, a new pre-treatment method needs to be developed to reduce matrix interference in future.

## Data Availability

The original contributions presented in the study are included in the article/Supplementary Material; further inquiries can be directed to the corresponding author.
