# Supplementary material for: A Species-Specific Strategy for the Identification of Hemocoagulase Agkistrodon halys pallas Based on LC-MS/MS-MRM
Source: Front Mol Biosci. 2022 May 30;9:831293. doi: 10.3389/fmolb.2022.831293 (PMC9196937; doi:10.3389/fmolb.2022.831293)
Supplement: Supplementary file 1 [file DataSheet1.docx]

Supplementary Material

Table S1 Precision of the established LC-MS/MS-MRM method.

| **#** | **Area (cps)** | **Calculated Conc. (ng/mL)** | **Dilution ratio** | **Sample Conc. (ng/mL)** | **Mean (ng/mL)** | **RSD**  **（%）** |
| --- | --- | --- | --- | --- | --- | --- |
| 1 | 1.29E+05 | 0.178 | 83.33 | 14.83 | 14.53 | 2.1 |
| 2 | 1.27E+05 | 0.175 |  | 14.58 |  |  |
| 3 | 1.25E+05 | 0.172 |  | 14.33 |  |  |
| 4 | 1.24E+05 | 0.171 |  | 14.25 |  |  |
| 5 | 1.24E+05 | 0.171 |  | 14.25 |  |  |
| 6 | 1.30E+05 | 0.179 |  | 14.92 |  |  |

Table S2 Repeatability of the the established LC-MS/MS-MRM method.

| **#** | **Area (cps)** | **Calculated Conc. (ng/mL)** | **Dilution ratio** | **Sample Conc. (ng/mL)** | **Mean (ng/mL)** | **RSD**  **（%）** |
| --- | --- | --- | --- | --- | --- | --- |
| 1 | 1.21E+05 | 0.167 | 83.33 | 13.92 | 14.40 | 3.2 |
| 2 | 1.25E+05 | 0.172 |  | 14.33 |  |  |
| 3 | 1.26E+05 | 0.173 |  | 14.42 |  |  |
| 4 | 1.22E+05 | 0.168 |  | 14.00 |  |  |
| 5 | 1.32E+05 | 0.182 |  | 15.17 |  |  |
| 6 | 1.27E+05 | 0.175 |  | 14.58 |  |  |

Table S3 Stability of the the established LC-MS/MS-MRM method.

| **Time**  **(h)** | **Area (cps)** | **Calculated Conc. (ng/mL)** | | **Dilution ratio** | **Sample Conc. (ng/mL)** | **Mean (ng/mL)** | **RSD**  **（%）** |
| --- | --- | --- | --- | --- | --- | --- | --- |
| 0 | 1.14E+05 | 0.157 | 83.33 | | 13.08 | 14.42 | 4.5 |
| 1 | 1.23E+05 | 0.170 |  |  | 14.17 |  |  |
| 2 | 1.29E+05 | 0.178 |  |  | 14.83 |  |  |
| 4 | 1.28E+05 | 0.176 |  |  | 14.67 |  |  |
| 8 | 1.30E+05 | 0.178 |  |  | 14.83 |  |  |
| 10 | 1.30E+05 | 0.179 |  |  | 14.92 |  |  |
| 24 | 1.26E+05 | 0.173 |  |  | 14.42 |  |  |

Table S4 Detection of Hemocoagulase Agkistrodon halas pallas stostes.

| **Batch number** | **Area (cps)** | **Calculated Conc. (ng/mL)** | **Dilution ratio** | **Sample Conc. (ng/mL)** |
| --- | --- | --- | --- | --- |
| 20190201 | 1.35E+05 | 0.184 | 83.33 | 15.33 |
| 20190202 | 1.26E+05 | 0.173 |  | 14.42 |
| 20190203 | 1.36E+05 | 0.186 |  | 15.50 |
